# Supplementary material for: Artificial intelligence for research capacity strengthening: Two reviews and a pathway to shift power in global health
Source: PLOS Digit Health. 2026 Apr 16;5(4):e0001302. doi: 10.1371/journal.pdig.0001302 (PMC13086336; doi:10.1371/journal.pdig.0001302)
Supplement: S1 Text — (DOCX) [file pdig.0001302.s001.docx]

**SUPPLEMENTAL MATERIAL: Artificial intelligence for research capacity strengthening: Two reviews and a pathway to shift power in global health**

**Authors:** Brian Wahl,^1^ Tiffany Nassiri-Ansari,^2^ Daniel D. Redpath,^3^ Pascale Allotey,^4^ Nina Schwalbe^2,4^

World Count: 3786

**Affiliations:**

1. Department of Epidemiology of Microbial Diseases, Yale School of Public Health, New Haven, United States
2. Spark Street Advisors, New York, United States
3. Institute for Global Health and Development, Queen Margaret University, Edinburgh, United Kingdom
4. Department of Sexual and Reproductive Health and Research, which includes the UN Special Programme of Research, Development and Research Training in Human Reproduction, World Health Organization, Geneva, Switzerland
5. O’Neill Institute for National and Global Health Law, Georgetown University, Washington, D.C., USA

**Corresponding authors:**

- Nina Schwalbe: Spark Street Advisors, 55 White Street, New York, NY 10013; [nschwalbe@ssc.nyc](mailto:nschwalbe@ssc.nyc); +1 917 226 2645
- Brian Wahl: Yale School of Public Health, 60 College Street, New Haven, CT 06510; [brian.wahl@yale.edu](mailto:brian.wahl@yale.edu); +1 917 969 2656

1. **Protocol**

***Objectives***

The objective of this project was to synthesize existing evidence on the use of AI for research capacity in low- and middle-income countries (LMICs) and to situate this evidence within the broader literature on decolonization and power in global health knowledge production. The project consisted of two linked components:

- Systematic review of primary studies describing AI-enabled research capacity strengthening
- Review of reviews examining structural and epistemic determinants of power and knowledge generation in global health

**Research questions**

Systematic Review:

- How has AI, including large language models and other machine learning approaches, been applied to strengthen public health or scientific research capacity in LMICs?
- What types of capacity strengthening (e.g., analytical, methodological, training, writing support) have been reported?
- What gaps exist in the current evidence base?

Review of Reviews:

- What themes related to decolonization, power asymmetries, and knowledge generation are identified in existing reviews on global health research?
- How do these themes inform the interpretation of AI-enabled capacity strengthening in LMIC contexts?

**Inclusion and Exclusion Criteria**

Systematic review inclusion criteria

- Peer-reviewed articles published between January 1, 2000, and December 31, 2024.
- Articles that describe the use of AI tools for research purposes.
- Articles that include primary data, program descriptions, or detailed reports relevant to research capacity strengthening.
- Articles published in English, French, Spanish, or Portuguese.

Systematic review exclusion criteria

- Articles describing AI for clinical tasks unrelated to research.
- Bibliometric analyses or articles using AI to evaluate research outputs rather than support research capacity.
- Commentaries without relevant descriptions of AI-enabled capacity strengthening.
- Articles not available in full text.

Review of reviews inclusion criteria

- Review articles addressing decolonization, knowledge production, epistemology, or power in global health.
- Reviews published in English, French, Spanish, or Portuguese.
- Reviews that explicitly discuss knowledge generation or research capacity.

Review of reviews exclusion criteria

- Reviews focused solely on clinical interventions or health system performance without reference to knowledge generation or power.

**Databases**

- PubMed (all languages)
- Scopus (all languages)
- SciELO (Portuguese and Spanish)

Additional sources included manual searches of reference lists and targeted searches of gray literature from AI and global health organizations.

**Languages**

- English
- French
- Spanish
- Portuguese

The initial search only focused on English. Additional languages were selected to reduce Anglophone bias and capture scholarship from the Americas, Africa, and Europe relevant to AI and research capacity.

**Screening Process**

All search results were imported into Covidence for centralized management, where duplicates were removed automatically and confirmed manually. Two reviewers independently screened each title and abstract against the predefined inclusion and exclusion criteria. Articles that met the criteria at this stage proceeded to full-text review, which was also conducted independently by the same two reviewers. Any disagreements during either phase were resolved through discussion and consensus, with no need for third-party arbitration. Screening for the systematic review and the review of reviews was conducted separately but followed the same procedures to ensure consistency and methodological transparency.

**Data Extraction**

Data extraction was performed independently by two reviewers using a structured template developed at the outset of the review. For each included article, reviewers extracted publication characteristics, study design or review type, descriptions of AI applications (for the systematic review), and key themes related to decolonization and knowledge generation (for the review of reviews). Reviewers also documented methodological limitations when reported by study authors or when apparent from the study design. Extracted information was compared for consistency, and any discrepancies were resolved through consensus. This approach ensured a transparent and reproducible synthesis of findings across highly heterogeneous sources.

1. **Search strategy for systematic review**

*Scopus - English*

("research capacity" OR "capacity strengthening" OR "capacity development" OR "capacity building")

AND

("artificial intelligence" OR "large language model" OR "generative AI" OR "AI")

*PubMed - English*

(("research capacity"[Title/Abstract] OR "capacity strengthening"[Title/Abstract] OR "capacity development"[Title/Abstract] OR "capacity building"[Title/Abstract])

AND

("artificial intelligence"[Title/Abstract] OR "large language model"[Title/Abstract] OR "generative AI"[Title/Abstract] OR "AI"[Title/Abstract]))

*PubMed – French*

(“capacité de recherche” OR “renforcement des capacités” OR “développement des capacités” OR “renforcement des capacités”)

AND

(“intelligence artificielle” OR “grand modèle de langage” OR “IA generative” OR “IA”)

*PubMed – Portuguese*

("capacidade de pesquisa" OR "fortalecimento de capacidades" OR "desenvolvimento de capacidades" OR "desenvolvimento de capacidades")

AND

("inteligência artificial" OR "modelo de linguagem ampla" OR "IA generativa" OR "IA")

*PubMed – Spanish*

("capacidad de investigación" OR "fortalecimiento de capacidades" OR "desarrollo de capacidades" OR "desarrollo de capacidades")

AND

("inteligencia artificial" OR "modelo de lenguaje extenso" OR "IA generativa" OR "IA")

*SciELO – Portuguese*

("capacidade de pesquisa" OU "fortalecimento de capacidades" OU "desenvolvimento de capacidades" OU "capacitação")

E

("inteligência artificial" OU "modelo de linguagem ampla" OU "IA generativa" OU "IA")

*SciELO – Spanish*

("capacidad de investigación" O "fortalecimiento de capacidades" O "desarrollo de capacidades" O "desarrollo de capacidades")

Y

("inteligencia artificial" O "modelo de lenguaje extenso" O "IA generativa" O "IA")

1. **Search strategy for review of reviews focused on decolonizing global health research**

*English*

(("Decolonization"[Title/Abstract]) OR ("Decolonisation"[Title/Abstract]) OR ("Decolonisation"[Title/Abstract]) OR ("Decolonizing"[Title/Abstract]) OR ("Decolonising"[Title/Abstract]))

AND

(("Knowledge generation"[Title/Abstract]) OR ("Knowledge management"[Title/Abstract]) OR ("Epistemology"[Title/Abstract]) OR ("Research capacity"[Title/Abstract]) OR ("Research capacity"[Title/Abstract]) OR ("Training"[Title/Abstract]) OR ("Research"[Title/Abstract]))

AND

(("Systematic review"[Title/Abstract]) OR ("Scoping Review"[Title/Abstract]) OR ("Literature review"[Title/Abstract]))

AND

(("Public health"[Title/Abstract]) OR ("Global health"[Title/Abstract]))

*PubMed - French*

("Décolonisation" OR "Décoloniser")

AND

("Génération de connaissances" OR "Gestion des connaissances" OR "Épistémologie" OR "Capacité de recherche" OR "Capacité de recherche" OR "Formation" OR "Recherche")

AND

("Revue systématique" OR "Revue de la portée" OR "Littérature Revue")

AND

("Santé publique" OR "Santé mondiale")

*PubMed – Portuguese*

("Descolonização" OR "Descolonizando")

AND

("Geração de conhecimento" OR "Gestão do conhecimento" OR "Epistemologia" OR "Capacidade de pesquisa" OR "Treinamento" OR "Pesquisa")

AND

("Revisão sistemática" OR "Revisão de escopo" OR "Literatura revisão")

AND

("Saúde pública" OR "Saúde global")

*PubMed – Spanish*

("Descolonización" OR "Descolonizando") AND ("Generación de conocimiento" OR "Gestión del conocimiento" OR "Epistemología" OR "Capacidad de investigación" OR "Formación" OR "Investigación") AND ("Revisión sistemática" OR "Revisión del alcance" OR "Literatura revisión") AND ("Salud pública" OR "Salud global")

*SciELO - Portuguese*("Descolonização" OU "Descolonizando")

E

("Geração de conhecimento" OU "Gestão do conhecimento" OU "Epistemologia" OU "Capacidade de pesquisa" OU "Treinamento" OU "Pesquisa")

E

("Revisão sistemática" OU "Revisão de escopo" OU "Literatura revisão")

E

("Saúde pública" OU "Saúde global")

*SciELO – Spanish*

("Descolonización” O "Descolonizando") Y ("Generación de conocimiento" O "Gestión del conocimiento" O "Epistemología" O "Capacidad de investigación" O "Formación" O "Investigación") Y ("Revisión sistemática" O "Revisión del alcance" O "Literatura revisión") Y ("Salud pública" O "Salud global")
