# Supplementary material for: Artificial intelligence for research capacity strengthening: Two reviews and a pathway to shift power in global health
Source: PLOS Digit Health. 2026 Apr 16;5(4):e0001302. doi: 10.1371/journal.pdig.0001302 (PMC13086336; doi:10.1371/journal.pdig.0001302)
Supplement: Attachment — Submitted filename: REV1_Response to reviewers.docx [file pdig.0001302.s004.docx]

Dear Dr. Charles B. Delahunt,

Thank you for sharing the editor’s and reviewers’ thoughtful comments related to our proposed manuscript: “Artificial intelligence for research capacity strengthening: Two reviews and a pathway to shift power in global health.” Thank you, as well, for your patience as we worked to respond to these comments that required a major revision, including re-running the search in multiple languages. Below are our responses to the editor and reviewer comments. Please let us know if you require any additional information. We look forward to hearing back from you.

Below are our point-by-point responses to each of the comments.

Best regards,

Brian (on behalf of the co-authors)

**Editor comments**

Good morning, and thank you for your patience (if applicable) while we rounded up reviews. The good news is that the reviews are well-informed and thoughtful.

Both reviewers raise important points, and I urge you to fully address them, even at the cost of some effort.

Other comments:
The restriction of the HIC-only, English-only search scope, raised by both reviewers, is ironic and somewhat meta, given the topic. Addressing this by expanding your search space to include non-HIC sources would substantially increase the credibility and value of the paper. I suggest that you enlist Spanish, French, and maybe Portuguese speakers to search and navigate non-English literature. For Spanish literature, please see this AAAS article: <https://www.science.org/content/article/latin-american-journals-are-open-access-pioneers-now-they-need-audience> . I believe SciELO is a good source.

- RESPONSE: We expanded our search to include English, French, Portuguese, and Spanish-language research within PubMed and Scopus. In addition, we also searched SciELO specifically for Portuguese and Spanish papers. The reviewer and editors were very right to point out this oversight. We are glad that this was pointed out—we found an additional paper in Spanish that is highly relevant. Thank you.

Re the distinction between isolated, single task uses of AI vs capacity-building uses of AI: Some of the AI uses you cite (eg lines 184-185, 208 - 209) appear to be cases where AI is used to tackle a particular research topic, which is a different thing from more general use to increase capacity. How does this affect your assessment?

- RESPONSE: We agree that AI applications fall along a spectrum from single-task research uses to broader, system-level capacity strengthening. In our revision, we clarified this distinction by differentiating between individual-level capacity strengthening (training researchers to use AI tools for discrete analytic, writing, or knowledge management tasks) and institutional or system-level capacity strengthening (initiatives that build sustained research infrastructure, governance, and autonomy). We added text in the Methods, Results, and Discussion to explicitly describe how we used this distinction in our analysis.

Also relevant: What countries originated the cited AI uses? Whether they involve HIC researchers or non-HIC researchers, this should be clarified because it bears directly on the main themes of the paper.

- RESPONSE: We agree that identifying whether the AI applications arose from HICs or LMICs would provide additional insight into power dynamics in global health knowledge production. However, systematically classifying author origin proved infeasible as authors from LMICs can be based at institutions in HICs and vice versa. Addressing this accurately would require direct author contact or independent verification, which was beyond the scope of the review.

Reviewer #1: The manuscript addresses an important and timely topic at the intersection of artificial intelligence (AI), global health (GH), and research equity. However, the central theoretical framing requires significant clarification and revision.

Major Comments:

1. Theoretical Inconsistencies and Lack of Clarity:

The manuscript begins by identifying a gap in agenda-setting and knowledge production and posits that AI may help build research capacity in low- and middle-income countries (LMICs), thereby shifting power dynamics in global health. However, the literature review suggests that these power imbalances are primarily due to the dominance of institutions in high-income countries (HICs) in setting research priorities, directing funding, and defining what counts as “valid” knowledge. The manuscript then pivots back to promoting capacity building via AI—without sufficiently addressing that the core issue may not be capacity gaps in LMICs, but rather inequities in funding flows and decision-making authority. This disconnect in theoretical framing weakens the central argument. The authors should reconcile these perspectives or reframe the theory to accurately reflect the root causes of power asymmetries in global health.

- RESPONSE: Thank you for this important observation. We agree that power asymmetries in global health are rooted in deeper structural inequities related to agenda-setting, funding flows, and the institutional dominance of HIC-based organizations. In response, we revised the Introduction to explicitly note that capacity strengthening, including AI-enabled approaches, cannot by itself address these structural determinants of power. We also added clarifying text in the Discussion to articulate that AI-supported capacity building must be situated within broader reforms aimed at shifting the political economy of knowledge production.

2. Scope of the Systematic Review:

The papers included in the systematic review are not all focused on LMICs. Of the three global papers cited, two originate from institutions based in HICs, and the third (Khan, 2023) includes a mixture of institutions, though the specific contributions of Northern versus Southern actors are unclear. This distinction matters, as the paper draws conclusions about the role of AI in LMICs based on existing infrastructures, which are often shaped by Northern-led institutions. Greater transparency is needed regarding the geographical and institutional origins of the reviewed studies, especially if conclusions are being generalized to LMIC contexts.

- RESPONSE: Thank you for this helpful point. We agree that understanding the institutional context of AI-related research is important for interpreting its implications for LMIC capacity strengthening. While we did not systematically classify the origin or identity of authors, the institutional addresses reported in several global papers suggest that many contributing teams were based in high-income country institutions. We added clarifying text in the Results and Discussion to acknowledge this pattern and to note that it reflects broader structural asymmetries in global health knowledge production.

3. Framing and Methodological Concerns:

The manuscript must make a clearer choice in framing: is it exploring what large language models (LLMs) can do to assist researchers in LMICs, or is it proposing a broader theory of AI-led shifts in power dynamics in global health? The current combination of both makes the theoretical and methodological structure feel underdeveloped. An additional "Review of Reviews" appears forced and isn’t adding much value to the overall theory.

- RESPONSE: We appreciate this thoughtful comment. We intended to connect empirical evidence on AI-enabled research capacity strengthening with the broader theoretical debates on power and knowledge production in global health. To make this framing more explicit, we clarified in both the Introduction that the review-of-reviews was included to provide the decolonial and structural context needed to interpret the findings of the systematic review.

Minor Comments:

\# Lines 72–75: The reference to SRHR appears abruptly. Consider explaining how this focus emerged or provide a clearer transition.

- RESPONSE: We have expanded here.

\# Lines 161–167: Please include relevant citations to support the claims made.

- RESPONSE: We have included.

\# Lines 174–175: The mention of "serious methodological limitations" is vague. Specify what these limitations are.

- RESPONSE: We have clarified here.

Reviewer #2: I would like to congratulate the authors on this review which tackles an important, under-explored question—how AI could rebalance research capacity and power between high- and LMIC. The dual-synthesis design (systematic review of primary studies plus umbrella review of de-colonisation literature) is conceptually strong, and of interest and the supplementary material now supplies full search strings and clear PRISMA flow charts. Nevertheless, several elements remain incomplete, limiting a little bit some of the methodological rigour and reproducibility.

- RESPONSE: Thank you for the supportive comments. We appreciate your encouragement.

First, no protocol was registered and none seems to be provided. PROSPERO registration is not strictly mandatory for publication, but it is considered best practice and from what this reviewer can tell it is encouraged by PLOS to protect against post-hoc decision-making; perhaps the authors should register retrospectively (or upload a dated protocol) and document any changes?

- RESPONSE: We agree that preregistration can strengthen methodological transparency. While we did not preregister this review, we have included the details of the protocol in the supplemental materials. This protocol outlines the review objectives, search strategy, and planned analytic approach. We hope this contributes to the transparency and reproducibility of this review.

Second, risk-of-bias and certainty assessments are entirely absent. The justification by the authors seems solid but even with heterogeneous study designs, the primary record should be appraised with an appropriate JBI/NIH tool, and the eight included reviews assessed with ROBIS or AMSTAR-2; overall certainty could then be graded with GRADE-CERQual. Without this step, the narrative synthesis rests on un-weighted evidence and its conclusions may over-reach. That being said, this reviewer is not an expert on systematic reviews and perhaps this is not disqualifying given the authors provided justification.

- RESPONSE: We considered applying formal risk-of-bias and certainty assessments; however, only two of the included papers contained primary empirical data, and the remaining studies were commentaries, descriptive reports, or narrative reviews for which validated appraisal tools (e.g., JBI, NIH, ROBIS, AMSTAR-2) are not applicable. Because these tools cannot be used consistently across the included study types, a structured assessment would have produced misleading or incomparable judgments. To address this, we strengthened the Methods section to clarify our rationale and added language noting that we extracted and described methodological limitations narratively to maintain transparency.

Third, the search remains limited to PubMed and Scopus, in English only. Extending to EMBASE, Web of Science, Global Health, IEEE Xplore and at least one regional database, plus a grey-literature/pre-print sweep, would reduce selection bias and better capture LMIC scholarship. Any language restriction must be justified.

- RESPONSE: Yes, we agree entirely. In response to reviewer and editor feedback, we substantially expanded the search to include French, Spanish, and Portuguese-language publications. We added SciELO as a regional database to better capture Latin American scholarship. We documented these expanded searches in detail in the supplemental materials.

Fourth, the Data-Availability statement says “NA”, which could contravene PLOS policy. De-duplicated citation lists, screening logs, extraction sheets and any analytic code should be deposited in an open repository (e.g., Zenodo or OSF) and the DOI cited.

- RESPONSE: All data associated with this review will be deposited in an open-access repository. We note that several other PLOS papers do not mention this. However, two wrongs do not make a right. We will upload our data accordingly.

Finally, minor editorial issues persist (line-numbers in the text, “World Count” header, duplicated Chen 2024 reference).

- RESPONSE: Thank you! We have addressed.

In sum, the manuscript is topical and potentially impactful, but it requires some revision to meet PLOS’ standards for transparency and methodological soundness as far as systematic reviews go. Addressing the four points above—protocol registration, risk-of-bias certainty appraisal (potentially) , expanded search strategy, and compliant data sharing—together with routine polishing, will substantially strengthen the work and lend credibility to its claims.

- RESPONSE: We appreciate your very helpful comments.
